# Core components of infection prevention and control programs at the facility level in Kazakhstan: key challenges and opportunities

**DOI:** 10.1186/s13756-023-01264-6

**Published:** 2023-06-22

**Authors:** Anna Deryabina, Ainur Aiypkhanova, Almat Juvashev, Kuanysh Alimbetov, Kanat Tekebayev, Getachew Kassa, Andrea A. Howard

**Affiliations:** 1 Mailman School of Public Health, ICAP at Columbia University, 34/1 Samal-3, 050051 Almaty, Kazakhstan; 2 Independent Public Healthth Consultant, Astana, Kazakhstan; 3 National Centre for Public Health of the Ministry, Astana, Kazakhstan; 4 Mailman School of Public Health, ICAP at Columbia University, New York, USA

**Keywords:** Infection prevention and control, WHO core components, Facility assessment, Kazakhstan

## Abstract

**Background:**

Kazakhstan is developing a National Roadmap to strengthen its Infection Prevention and Control (IPC), but until recently has lacked a country-wide facility-level assessment of IPC performance gaps.

**Methods:**

In 2021, the World Health Organization (WHO)’s IPC Core Components and Minimal Requirements were assessed at 78 randomly selected hospitals across 17 administrative regions using adapted WHO tools. The study included site assessments, followed by structured interviews with 320 hospital staff, validation observations of IPC practices, and document reviews.

**Results:**

All hospitals had at least one dedicated IPC staff member, 76% had IPC staff with any formal IPC training; 95% established an IPC committee and 54% had an annual IPC workplan; 92% had any IPC guidelines; 55% conducted any IPC monitoring in the past 12 months and shared the results with facility staff, but only 9% used monitoring data for improvements; 93% had access to a microbiological laboratory for HAI surveillance, but HAI surveillance with standardized definitions and systematic data collection was conducted in only one hospital. Adequate bed spacing of at least 1 m in all wards was maintained in 35% of hospitals; soap and paper towels were available at the hand hygiene stations in 62% and 38% of hospitals, respectively.

**Conclusions:**

Existing IPC programs, infrastructure, IPC staffing, workload and supplies present within hospitals in Kazakhstan allow for implementation of effective IPC. Development and dissemination of IPC guidelines based on the recommended WHO IPC core components, improved IPC training system, and implementation of systematic monitoring of IPC practices will be important first steps towards implementing targeted IPC improvement plans in facilities.

**Supplementary Information:**

The online version contains supplementary material available at 10.1186/s13756-023-01264-6.

## Background

Inadequate infection prevention and control (IPC) practices in healthcare facilities is the main driver of increasing rates of antimicrobial resistance (AMR) and healthcare-associated infections (HAIs) [1–3] and is a growing public health concern worldwide [4]. Studies estimate that one in 25 hospitalized patients in the United States and one in 18 hospitalized patients in Europe has a HAI on any given day [5, 6]. The current SARS-CoV-2 pandemic underscores the need for adequate IPC systems in healthcare facilities [7].

Kazakhstan is an upper-middle income country in Central Asia with a population of around 18 million. According to the 2021 Global Health Security Index report, there is insufficient evidence that the national public health system in Kazakhstan monitors for and tracks the number of HAI at the facility-level [8]. In 2022, with support from the World Health Organization (WHO), Kazakhstan will conduct the first-ever point-prevalence survey of HAIs. While data on HAIs in Kazakhstan published in international journals are scarce, several studies suggest transmission of HAIs in hospitals is an important problem [9–14]. In 2006, Kazakhstan experienced a transfusion-related HIV outbreak among children [15]. Kazakhstan has a high prevalence of both hepatitis B (HBV) and hepatitis C (HCV) [16], with dental surgery, blood transfusions, and frequent injections identified as factors associated with increased odds of HCV seropositivity [17, 18].

Transmission of AMR in healthcare settings and HAIs can be prevented through comprehensive and robust IPC programs [19–21]. The WHO released evidence-based guidelines on core components of IPC at the facility level in 2016 [21, 22]. These guidelines cover eight components of IPC listed below in Table 1 and include 14 recommendations and best practice statements:

**Table 1:** Core IPC components and WHO recommendations

Core component	Key WHO recommendations
1. IPC programmes	An IPC programme with a dedicated, trained team should be in place in each acute health care facility for the purpose of preventing HAI and combating antimicrobial resistance (AMR) through IPC good practices
2. IPC guidelines	Evidence-based guidelines should be developed and implemented for the purpose of reducing HAI and AMR. The education and training of relevant health care workers on the guideline recommendations and the monitoring of adherence with guideline recommendations should be undertaken to achieve successful implementation
3. IPC education and training	IPC education should be in place for all health care workers by utilizing team- and task-based strategies that are participatory and include bedside and simulation training to reduce the risk of HAI and AMR
4. Surveillance	Facility-based HAI surveillance should be performed to guide IPC interventions and detect outbreaks, including AMR surveillance with timely feedback of results to health care workers and stakeholders and through national networks
5. Multimodal strategies	IPC activities using multimodal strategies should be implemented to improve practices and reduce HAI and AMR
6. Monitoring/audit of IPC practices and feedback	Regular monitoring/audit and timely feedback of health care practices according to IPC standards should be performed to prevent and control HAI and AMR at the health care facility level. Feedback should be provided to all audited persons and relevant staff
7. Workload, staffing and bed occupancy	The following elements should be adhered to in order to reduce the risk of HAI and the spread of AMR: (1) bed occupancy should not exceed the standard capacity of the facility; (2) health care worker staffing levels should be adequately assigned according to patient workload
8. Built environment, materials and equipment for IPC at the facility level	Patient care activities should be undertaken in a clean and/or hygienic environment that facilitates practices related to the prevention and control of HAI, as well as AMR, including all elements around the water, sanitation, hygiene (WASH) infrastructure and services and the availability of appropriate IPC materials and equipment. Materials and equipment to perform appropriate hand hygiene should be readily available at the point of care

In Kazakhstan, several decrees of the Ministry of Health (MoH) exist that describe facility-level IPC requirements and are treated as National IPC guidelines. However, the decrees are fragmented and only provide general IPC recommendations. In 2022, the MoH expects to finalize and approve updated National IPC guidelines. In 2022, the MoH also plans to develop and approve a comprehensive National Roadmap to strengthen its IPC Program. To provide identify existing gaps in IPC at the health facility level and inform development of the Roadmap, ICAP at Columbia University in close collaboration with the National Center for Public Health (NCPH) of the MoH conducted a systematic cross-sectional assessment of IPC practices in a sample of general multispecialty hospitals in Kazakhstan.

## Methods

### Study design, hospital selection and recruitment

A random sample of 80 hospitals participating in the National Social and Health Insurance system was selected from all geographical regions of Kazakhstan, stratified by service status (i.e. public/private and urban/rural), with probability proportional to size. Hospitals providing only psychiatric services or tuberculosis treatment were excluded because of the specialized care they provided and unique IPC issues they faced. To recruit hospitals, the NCPH contacted each facility manager in writing to inform them about the assessment and invite them to participate. Facility managers were encouraged to participate but assured that declining would not affect their employment in any way. If a facility manager agreed to participate, the NCPH and ICAP scheduled an assessment visit, and the manager identified members of the hospital IPC team (e.g., IPC Focal Persons, doctors, nurses, epidemiologists) that were invited to a meeting with the assessment team. Upon arrival, the assessment team conducted a short introductory meeting with the facility management and the hospital IPC team to inform them about the assessment procedures and feedback process and to obtain verbal informed consent from each potential participant.

### Pilot study and data collection

The assessment tool was based on the IPC Assessment Framework (IPCAF) on Core Components of IPC and the recommendations included in the WHO “Guidelines on Core Components of Infection Prevention and Control Programs at the National and Acute Health Care Facility Level” [23]. The IPCAF is primarily intended to be used by facilities as a self-assessment tool but can also be successfully used for the purpose of joint external assessments [24–26]. A study published in 2020 highlights that effective utilization of the IPCAF tool requires a deep understanding of the WHO terminology and underlying concepts to avoid misinterpretation and misreporting of data [27]. To improve data quality and avoid biased reporting, the team conducted three meetings with local IPC specialists to review and revise the questions to make them more relevant to Kazakhstan, and add additional questions that elicited additional details or verification. The modified questionnaire (Additional file 1: Annex 1) was then transferred into ICAP’s online survey data collection system (e-Survey) and piloted at two hospitals located in the capital Nur-Sultan that were not included in the study sample. Results of the pilot were used to revise the questionnaires and data collection procedures.

Data were collected during August–September 2021 by a team of local specialists involved in IPC implementation, monitoring, and training. All data collectors were trained by ICAP at Columbia University in protocol implementation, interviewing techniques and ethical considerations. All hospital assessments were conducted during a 2-day visit by two study team members. The first part of the assessment consisted of: (1) individual and small group structured key informant interviews, conducted in Russian, with hospital managers and facility IPC team members, and (2) a review of the facility’s IPC-related documents. Discrepancies in responses to the same question by different participants from the same hospital were resolved by facilitating a discussion among hospital IPC team members until a final answer was agreed upon and recorded. During the second part of the assessment, the study team conducted a facility walk-through using observations to verify answers provided during the interviews. In case of any discrepancies between information provided during the interviews and observations during the facility walk-through, data from different methods were discussed the facility staff to ensure facilities understand the differences and reported separately. Data were entered into a tablet computer using e-Survey. Answers to open-ended questions were audio-recorded and then transcribed for analysis. At the end of the assessment, the study team shared its constructed feedback with an IPC team at each hospital and provided the team with copies of WHO Guidelines on Core Components of IPC Programs at the National and Acute Health Care Facility Level to guide their quality improvement efforts.

### Data analysis and reporting

Descriptive analysis was conducted for categorical data using frequencies and cross-tabulation. Qualitative data from key informant interviews were grouped into meaningful patterns and/or themes through content and thematic analysis using NVivo^©^. Data from individual interviews were either linked with data from the document review and facility observations to allow for multidimensional descriptions of IPC core components at the facility level or integrated with each other to produce a more comprehensive picture of IPC core components at the facility level [28]. The study findings were reported in line with STROBE (Strengthening the Reporting of Observational Studies in Epidemiology) guidelines [29]. A final written report was shared with the Ministry of Health and all the hospitals that participated in the assessment. Summary results were presented during the National IPC Conference conducted in September 2022.

### Ethics

The protocol was approved by the Institutional Review Boards at the Astana Medical University of the Ministry of Health of Kazakhstan (the National Ethics Committee) and Columbia University Medical Center. Participation in the study was voluntary and all participants provided verbal informed consent prior to participation, with the option to withdraw consent at any time. Participants were informed that results of the assessment would be presented to the MoH in the form of a summarized report with no data on individual hospitals included. No compensation for participation was provided.

## Results

Eighty hospitals were included in the sample, of which two private hospitals refused to participate. Of the 78 participating hospitals, including 70 urban hospitals (45 were public and 25 were private), and 8 were rural hospitals (all public), representing approximately 9.5% of all hospitals in Kazakhstan. The median bed capacity was 171 (interquartile range (IQR) 69–320) beds per facility. A total of 320 people were interviewed, including 125 facility managers and 195 IPC team members.

Key assessment findings related to facility-level IPC system characteristics as recommended by WHO are summarized in the text below. Detailed assessment results are presented in Additional file 2: Annex 2.

### IPC program components

All hospitals included in the assessment had at least one designated IPC specialist, whose primary role and direct responsibility included organization, coordination, and monitoring of IPC practices, but only 59 (76%) hospitals had at least one IPC team member who had received formal IPC training. Twenty-four (31%) hospitals had only one designated IPC specialist that was formally trained, including 8 hospitals where IPC focal point responsibilities were designated to a physician specifically trained in IPC, and 16 hospitals that had a designated and trained IPC nurse. Thirty-one (40%) hospitals had more than one properly trained IPC team member. Overall, nurses specially trained in IPC were engaged in organization, coordination, and monitoring of IPC practices at 48 (61%) hospitals.

Most hospitals (n = 71, 91%) had a document describing the facility's internal IPC policy, but only 4 (5%) had implemented all WHO-recommended elements of IPC programs including clearly defined objectives based on local epidemiology, annual IPC workplans, adequate improvement measures and targets, and a specified IPC budget.

The majority (n = 74, 95%) of hospitals surveyed had an established multidisciplinary IPC committee that advises the IPC team. Seventy-three (94%) hospitals reported having senior leadership (e.g., administrative director, chief executive officer, medical director) or senior clinical staff (e.g., chief physician, chief of nursing) included in their IPC committee. Additionally, IPC committees at 30 (38%) hospitals were comprised of a multidisciplinary group that included facility management staff, such as biosafety and WASH staff. Sixty (77%) hospitals reported that the committee met at least once in the past 12 months. However, documentation of all IPC committee meetings, as evidenced by meeting notes, was only available only in 70% (42/60) of these hospitals.

Seventy-five (97%) hospitals had access to a microbiology laboratory within or outside of the facility for day-to-day use.

### IPC guidelines

Seventy-two (92%) hospitals had IPC guidelines available, including 37 (47%) that used national guidelines, and 35 (49%) that implemented internal guidelines developed by their own facility staff based on national and/or international guidelines. Most hospitals had some guidelines and/or SOPs on hand hygiene (70 hospitals, 90%), disinfection and sterilization (69 hospitals, 88%), and waste management (69 hospitals, 88%). Only 37 hospitals (47%) had SOPs on screening for SARS-CoV-2 of the incoming patients (triage and patients flow arrangement) and only 34 hospitals (44%) had standard operating procedures (SOPs) on transmission-based precautions. Overall, 62 (79%) hospitals used various means of dissemination for newly developed/revised SOPs and guidelines, including posting them on information boards available for all employees, announcing newly-developed SOPs at regular hospital meetings, and/or conducting training sessions with or without interactive materials. Fifty (64%) hospitals reported training clinical staff on the IPC guidelines during interactive and/or non-interactive sessions.

### IPC training

In this assessment, IPC trainings included all educational and/or skills building sessions that covered any IPC procedures and practices. Sixty-four (82%) hospitals had conducted IPC trainings in the previous 12 months. Although most hospitals trained clinical and non-clinical staff on IPC, ongoing annual IPC training for clinical staff was formally required (e.g. mandated by an internal policy) at 53 (68%) hospitals.

Fifty-five (71%) hospitals conducted IPC trainings for all clinical staff as part of new employee orientation in addition to mandatory refresher trainings at least annually.

Forty-four (56%) of 78 hospitals conducted IPC trainings for all non-clinical staff during orientation as well as regular mandatory refresher trainings at least annually. During individual interviews, IPC managers and hospital staff at 46 (58%) hospitals mentioned the lack of regular IPC training for clinicians and IPC staff as one of the key challenges to implementing IPC.

### HAI surveillance

Fifty-eight (74%) hospitals reported conducting HAI surveillance, and 73 (93%) hospitals had access to a microbiological laboratory for HAI surveillance purposes. However, only one hospital had all HAI surveillance components recommended by WHO including a list of priority HAIs, standardized case definitions, standardized data collection and review methods, and clearly defined roles and responsibilities. During individual interviews, facility managers and staff at 53 (68%) hospitals mentioned punitive sanctions, including monetary fines, from the control authorities, fear of punishment and unwillingness of public disclosure were the main barriers to effective HAI surveillance. Limited training of healthcare workers in HAI surveillance and lack of clear guidelines and data collection tools were also noted as barriers to effective IPC implementation by respondents in 13 (13%) and 9 (9%) hospitals, respectively.

### Multimodal strategies

Facility use of multimodal strategies for hand hygiene and injection safety were assessed. Injection safety was specifically targeted given the high prevalence of hepatitis C in Kazakhstan. While 75 (96%) hospitals reported having reminders, posters, or other tools to promote hand hygiene, only 25 (32%) hospitals displayed them at all hand hygiene stations. Six (8%) hospitals used additional methods such as thematic conferences and multidisciplinary ward rounds to improve team communication for hand hygiene across units. Four (5%) hospitals reported having reminders, posters, or other tools to promote injection safety, yet only three (4%) had visible reminders, posters, or other tools to raise awareness of injection safety at all stations. Managers showed visible support and served as role models for hand hygiene and injection safety in 68 (87%) and 44 (56%) hospitals, respectively.

### IPC monitoring and audit

Twenty-three (29%) hospitals had an IPC monitoring/audit plan available, however only one of these plans had all of the necessary elements, such as clear goals and objectives, tools to systematically collect data, clearly defined roles and responsibilities, and a work plan or schedule. Thirty-five (45%) facilities had not conducted any structured IPC monitoring in the past 12 months. Thirty-four (44%) hospitals reported conducting internal monitoring/audits in the last 3 months, and thirty-two (94%) provided documentation of these monitoring/audits. Although none of the hospitals conducted internal monitoring/audits at least once a month for each category of IPC practices, eight hospitals conducted monthly routine internal monitoring/audits on at least 3 categories of IPC practices. Categories of IPC practices included: hand hygiene, intravascular catheter insertion and/ or care, wound dressing change, transmission-based precautions and isolation to prevent the spread of multidrug resistant organisms, cleaning of the ward environment, disinfection and sterilization of medical equipment/instruments, consumption/usage of alcohol-based handrub or soap, consumption/usage of antimicrobial agents, and waste management. Only 8 (10%) hospitals conducted and documented monitoring/audit in the past 12 months and shared the results with all cadres of facility staff, including clinical and non-clinical staff, IPC committee members and facility management.

### Workload, staffing and bed occupancy

Most (n = 70, 90%) hospitals had a system for responding to an increase in staff workload, either because of a decrease in the number of the healthcare workers or an increase in the number of the patients admitted to the facility. The coordination of all issues related to such situations was in most cases the responsibility of the hospital leadership. During observations, only a few hospitals (4%) had the patients placed outside the hospital wards. However, only 27 (35%) hospitals had adequate spacing (at least one meter) between beds in all units.

### IPC infrastructure and supplies

Most hospitals reported having the basic infrastructure and supplies needed to conduct IPC, including an adequate amount of hygiene supplies in stock (soap, towels, sanitizers) in 54 (79%) hospitals, and adequate level of decontamination and disinfection products (mops, detergents, buckets, disinfectants) in 71 (91%) hospitals. However, during facility-level observations, the study team observed that only 10% of hospitals had alcohol-based hand rub at each point of care, soap was available at the hand hygiene stations in all points of care in only 48 (62%) hospitals. Similarly, 57 (73%) hospitals reported having a supply of paper towels for at least 1 month, but only 30 (38%) had paper towels at the hand hygiene stations at all points of care.

### Key IPC challenges

All facility managers and IPC staff who participated in the study were asked an open-ended question regarding the key IPC challenges faced by their facility. A total of 180 responses were grouped into six main categories as outlined in Table 2 below.

**Table 2:** Key IPC challenges as reported by facility managers and IPC staff interviewed (N = 180)

Response category	IPC challenges	Number of respondents (% of the overall of response)	Illustrative quotes
Lack of sufficient qualified staff	Lack of properly trained IPC specialists; Insufficient trainings of IPC staff; Overload of IPC staff with work that is not directly related to IPC; Low salaries of IPC staff; Staff turnover; Limited opportunities for IPC staff development and lack of mentoring support and technical assistance to facility-level staff in IPC-related issues; Low level of IPC knowledge among clinical staff	79 (44)	“there is no technical support [for IPC] and very limited training opportunities for the IPC team and other facility staff, not enough informational materials on IPC” “the existing medical education system is suboptimal. Graduates of public health departments have no understanding of key IPC principles and only limited knowledge of infectious diseases” ‘we don’t have an in-house full-time designated IPC specialist” “epidemiologists are one of the lowest paid staff in the hospital”
Insufficient integration of the IPC issues into overall quality control of the health services provided	Low commitment to IPC issues by hospital management and staff; Low involvement of other specialists in improving IPC work; Lack of systematic work to improve IPC Lack of systematic work to improve IPC	50 (28)	‘clinicians do not demonstrate enough commitment to improve IPC practices and are not motivated to comply with IPC requirements” “we [IPC team] do not have direct communication with the hospital leadership, there is also poor collaboration with clinicians” “we don’t get any support from the quality improvement department”
Inadequate infrastructure of hospitals	Old hospital buildings requiring renovation; Lack of mechanical ventilation and central sewerage; Inability to ensure appropriate flow of patients and healthcare workers due to inappropriate building design, especially at older hospitals; Lack of in-house microbiological laboratory Poor quality of microbiological support	45 (25)	“our building needs to capital improvements, but we can’t even afford to complete routine renovations” “because of poor building conditions we cannot get approval to open a laboratory at our hospital” “we don’t have any mechanical ventilation in our hospital” “our hospital is located in an old building that was not initially designed and built as a hospital”
Inadequate funding for IPC activities	Limited funding to procure PPE and disinfectants Lack of designated budget for IPC needs Very complex state procurement procedures that delay procurements	15 (8)	“our budget for IPC is insufficient” “we don’t always have enough funds to procure hand sanitizers and all the PPE” “The amount of reimbursement hospitals receive [from the state socio-medical insurance fund] for each treated case does not include any IPC-related costs, and we don’t have a separate budget designated budget for IPC; IPC is always funded on residual basis.”
Outdated IPC regulations	Inconsistency of national regulations with the most updated international IPC recommendations; Inconsistency between different national regulations; Punitive policies, including fines for HAIs	8 (4)	“fear of fines and sanctions [from the state sanitary-epidemiological committee]” “national policies are not in line with the WHO recommendations, no SOPs on many [IPC] practices” ‘hospital do not collect or report information of HAIs because they fear sanctions and decreased reimbursements from the insurance fund”
Limited access to timely and quality laboratory results	No access to in-house laboratory Delayed return of lab results Inconsistency between clinical manifestations and laboratory results	7 (4)	“results that we get from the laboratory are always delayed and often do not match the clinical picture”

## Discussion

This study is the first formal and systematic assessment of IPC core components among a large sample of multi-specialty hospitals in Kazakhstan. Including nearly 10% of all hospitals in Kazakhstan [30], the study provides a summary of existing facility-based IPC systems in Kazakhstan and highlights several priority areas for improvement so that the systems comply with core IPC components recommended by WHO. Many gaps in core IPC components identified during this study were also noted during a situational assessment of national-level IPC for maternity and newborn medical services in Kazakhstan conducted by the National Center for Public Health with assistance from the United Nations Population Fund in 2019 [31].

Specifically, we found challenges related to IPC training for IPC focal points and healthcare workers in general. All of the hospitals surveyed had at least one designated IPC specialist, whose direct responsibility was to organize, coordinate and monitor IPC practices. Despite recommendations for all IPC teams to include IPC nurses [32], 13% of hospitals did not have any IPC nurses. In only 72% of hospitals had IPC focal points completed any formal certified training in IPC. During individual interviews, IPC focal points also mentioned limited opportunities for IPC staff to receive IPC guidance and mentoring, and to share experience with IPC staff in other hospitals. Implementation of regular IPC trainings that include interactive skill demonstration sessions for all facility staff that encounter patients and wards are important to ensure compliance with IPC practices [22, 33]. While most hospitals (71%) provided briefing and training in IPC to healthcare workers at the time of recruitment, only a half of the hospitals required that all healthcare workers complete IPC training annually. Very few IPC trainings conducted by hospitals during 12 months prior to the survey included interactive skills demonstration session. Suboptimal IPC training at all levels of medical education have also been reported by other studies in Kazakhstan [34–36]. Similar shortcomings were demonstrated in other countries in the Eastern Europe, Caucuses, and Central Asia region [25, 37], as well as other parts of the world, including high-income countries [38–41].

Data for this study were collected during the second year of the SARS-CoV-2 pandemic. Standard precautions as they apply to the prevention of SARS-CoV-2, as well as transmission-based precautions, are essential in the reduction of SARS-CoV-2 transmission in healthcare settings [7]. While most hospitals had some guidelines and/or SOPs on hand hygiene (90%), only 55% of hospitals had any SOPs on transmission-based precautions while providing care to suspected or confirmed cases of COVID-19 and only 47% had SOPs on screening for SARS-CoV-2 of the incoming patients (triage and patients flow arrangement). Also, the SARS-CoV-2 pandemic has clearly demonstrated the need for enhanced IPC practices to avoid the threat of ventilator and non-ventilator-associated hospital-acquired pneumonia as one of the most common and morbid HAIs [42]. Only a very small proportion of hospitals (14%) had any guidelines and/or SOPs on prevention of hospital-acquired pneumonia. It is, however, important to note that this challenge is also not unique to Kazakhstan, as non-ventilator associated pneumonia is not tracked, reported, or actively prevented by many hospitals around the world [43]. According to the WHO, prevention and management of infections caused by multi-drug resistant (MDR) gram-negative pathogens is considered as a high priority health threat globally, including in Kazakhstan [14]. At the time of the survey, there was no national guidance on the use of antibiotics in healthcare practices in Kazakhstan, and facilities are expected to develop their own guidelines [44]. Our results show that guidelines and SOPs on prevention of MDR pathogens were available only at 10% of hospitals. Limited availability of guidelines on antibiotic stewardship and prevention of MDR pathogens was also noted in facility-level studies conducted in other countries, such as Georgia and Korea [24, 25].

Although 45% of hospitals reported conducting routine HAI surveillance, only one hospital had an HAI surveillance system that included all of the key WHO recommendations. A global survey of national-level IPC core components published in 2021 showed that less than half of the 88 countries surveyed had established national surveillance networks on HAIs [45]. HAI surveillance also had the lowest scores among low income countries in the first WHO global survey assessing IPC program implementation at the facility level using IPCAF [27].

Regular monitoring of IPC practices and timely feedback to all relevant staff is critical to prevent and control HAI at the facility-level (WHO, 2016). Many studies from different settings have demonstrated improvements in universal precautions compliance and reduction in HAIs after implementation of multimodal strategies that include routine observation, feedback, and promotion of effective IPC practices [46–50]. In our study very few hospitals used a systematic approach to routine monitoring of IPC practices, including hand hygiene and injection safety, which is similar to the situation in facilities in low-income countries around the world [27].

The provision of sufficient space in clinical areas, particularly for each bed space, is one of the most important considerations in the planning and design of inpatient accommodation [51]. Overcrowding increases transmission of HAIs, including MDR pathogens, in hospital settings [52, 53]. Therefore, an adequate spacing (of at least 1 m) between beds in all units is recommended by the WHO and is also required in accordance with the national regulations. However, only 35% of hospitals in the study met that criterion. A similar situation with suboptimal spacing between hospitals beds was observed in 2009 in the neighboring republic of Kyrgyzstan [54].

Patient care activities should be undertaken in a clean and/or hygienic environment that facilitates practices related to the prevention and control of HAI, as well as AMR, including all elements around the WASH infrastructure and services and the availability of appropriate IPC materials and equipment. Materials and equipment to perform appropriate hand hygiene should be readily available at the point of care [22]. Results of this assessment show very low availability that only 10% of hospitals had alcohol-based hand hygiene supplies at points of care. Hand hygiene is recognized as a leading cause of HAI, therefore, improving availability of hand hygiene supplies combined with improved training rates and only 33% with single-use towels at each sink and a more systematic approach to monitoring and reporting of IPC practices are urgently needed to improve prevention and control of HAIs in Kazakhstan [27, 55].

This study has several limitations. Only two private hospitals refused participation in the study, which could indicate a hesitancy, especially among government-funded hospitals, to decline participation in a study endorsed by the Ministry of Health. Although all the hospital managers and IPC staff were informed that participation in the assessment is voluntary and assured that declining will not affect their employment in any way, and that only the aggregate results will be reported, there still could have been respondent bias leading to underreporting of existing malpractices or shortcomings.

The lack of understanding of certain concepts, such as “multimodal strategies” and “methodology for calculating HAI”, by the healthcare workers could also affect the ability of the respondents to provide accurate information. To the extent possible, all questions were explained and clarified, and respondents' answers during the interviews were verified by checking supporting documentation and observations. Data collection was carried out by a team of the specialists from the NCPH and external IPC specialists. All specialists involved in data collection received a 2-day training on the basic WHO IPC recommendations and the protocol and tools for the situational analysis. Most of the questions for the hospitals were structured and included validation through observation and/or document review. In addition, each hospital had a team of two specialists, with the composition of the teams changing over the course of the assessment to avoid distortion of the information obtained as much as possible. Nevertheless, it cannot be completely excluded that differences in answers to some questions between inpatient health facilities were due to different understanding of the questions by the data collection specialists and different interpretation of the answers. The assessment only included hospitals that participated in the National Social and Health Insurance system, which constitute approximately 75% of all the hospitals in Kazakhstan. This could limit generalizability to hospitals that do not participate in the system. Lastly, although many of the survey questions were similar to the IPCAF questionnaires, the response options were different and thus direct comparison to other studies may not be possible.

## Conclusions

Our study shows that most of the hospitals in Kazakhstan that were surveyed have parts of an effective IPC program, namely an IPC committee, designated staff to organize, coordinate and monitor IPC, and basic infrastructure and supplies in place. Key challenges included the lack of sufficient qualified IPC staff and suboptimal training of clinical staff in IPC. To translate IPC programs into functional IPC activities, hospitals need to invest in building the capacity of their IPC teams, ensure routine IPC trainings for facility staff, and implement systematic and routine monitoring of IPC practices, as well as HAI surveillance. To make IPC programs comprehensive, hospitals need to clearly define their IPC objectives, develop annual IPC workplans, and implement adequate IPC improvement measures and targets.

## Supplementary Information

**Additional file 1**. Annex 1.

**Additional file 2**. Annex 2.

## Data Availability

The authors are responsible for the data described in the manuscript and assure full availability of the study material upon request to the corresponding author.

## Abbreviations

AMR: Antimicrobial resistance
DQA: Data quality audit
HAI: Health care-associated infections
ICAP: Center at the Mailman School of Public Health, Columbia University
IPCAF: IPC Situational analysis Framework
IPC: Infection prevention and control
MoH: Ministry of Health
MDR: Multidrug resistant
NCPH: National Center for Public Health
SOP: Standard operating procedure
WASH: Water, sanitation, and hygiene
WHO: World Health Organization
