# Supplementary material for: Core components of infection prevention and control programs at the facility level in Kazakhstan: key challenges and opportunities
Source: Antimicrob Resist Infect Control. 2023 Jun 22;12:59. doi: 10.1186/s13756-023-01264-6 (PMC10286477; doi:10.1186/s13756-023-01264-6)
Supplement: Supplementary file 1 — Additional file 1. Annex 1. [file 13756_2023_1264_MOESM1_ESM.docx]

# Annex 1. Guide to Semi-Structured Interviews with IPC Team – in-patient facilities

| VARIABLE ID | QUESTION | RESPONSE OPTIONS |
| --- | --- | --- |
| **Facility Background Information** | | |
| DISTRICT | District/Province/County | **Select from the drop-down list** |
| FACILITY | Facility name | **Select from the drop-down list** |

| **Data collector** (name) **___________________________**  **Number of people interviewed:**  **Health facility managers ___________________**  **IPC Team members _________________**  **Date of assessment:_______________**  **Start time: ______________ End time:_________** |
| --- |

**Step 1: Introduce all the assessment team members and provide a short introduction**

| VARIABLE ID | QUESTION | RESPONSE OPTIONS |
| --- | --- | --- |
| **Facility Background Information** | | |
| BEDS | Number of inpatient beds (excluding delivery beds) |  |
| BEDS_ICU | Of the total number of inpatient beds, how many are ICU beds? |  |
| CENSUS | Average inpatient census (if available) |  |
| ADMISSION | Number of inpatient admissions in 2020 (excluding delivery only admissions) |  |
| OUTPATIENT | Number of outpatient visits in 2020 |  |
| SURG | Number of surgeries performed in 2020 |  |
| DOCTOR_NUM | Number of medical doctors/clinical officers |  |
| NURSE_NUM | Number of registered nurses |  |
| LAB_NUM | Number of laboratory technologists/technician |  |
| PHARM_NUM | Number of pharmaceutical technologists/technician |  |
| CLEAN_NUM | Number of cleaning staff |  |

| **#** | **Question** | **Answer** |
| --- | --- | --- |
| **Core component 1: Infection Prevention and Control (IPC) Program** | | |
| 1 | Does your organization have an Infection Prevention and Control (IPC) plan or strategy?  **Choose one answer**  **Review a copy of the IPC plan** | 1. No **(skip to 2)** 2. Yes |
| 1A | Does the existing IPC programme have annual work plan for 2021?  **Choose one answer** | 1. No, there is an IPC plan/program but no time-bound objectives for this year 2. Yes, the existing plan/strategy has annual work plan for 2021 |
| 1B | Does the existing IPC programme have clearly defined IPC measurable outcome indicators for the facility?  **Choose one answer** | 1. No **(skip to 2)** 2. Yes, IPC objectives only 3. Yes, IPC objectives and measurable outcome indicators (that is, adequate measures for improvement. For example: KPI for IPC) 4. Yes, IPC objectives **and** measurable outcome indicators **and** set future targets |
| 1C | Are IPC objectives for the facility based local epidemiology and priorities according to risk assessments  **Choose one answer** | 1. IPC objectives based on local epidemiology and priorities according to risk assessments 2. No, there is no evidence to confirm that IPC objectives are based on local epidemiology and priorities according to risk assessments |
| 1D | Does your facility have a budget specifically allocated to the IPC programme (e.g. to address IPC materials, administrative support, staff)?  **Choose one answer** | 1. No 2. Yes 3. Don’t know |
| 2 | How many staff certified staff (nurses and/or doctors and/or epidemiologists and/or others trained in a certified IPC course) have responsibilities to work specifically on IPC activities?  **Please ask for a copy of the Terms of Reference for the Team or the IPC Focal point and the document that certifies their appointment (prikaz, training certificates).** | ____ staff positions that have IPC responsibilities **filled** by staff that **were not** trained in a certified IPC course, including  ___ nurses  ___ doctors  ____ staff positions that have IPC responsibilities **filled** by staff that **were trained** in a certified IPC course  ___ nurses  ___ doctors |
| 2A | What IPC professional development options were available for the IPC team members at your facility during the past 12 months? | 1. No professional development offered 2. IPC members attend IPC-related conferences or workshops 3. IPC members attend IPC-related training courses |
| 3 | Do you have an IPC Committee actively supporting the IPC team?  **Together review the order approving IPC committee membership, terms of work** | 1. No **(skip to 4)** 2. Yes |
| 3A | Which, if any, of the following professional groups are represented or included in the IPC Committee or an equivalent?  **SELECT ALL THAT APPLY**  **Please verify the answer based on the available documents.** | 1. Facility management (e.g. administrative director, chief executive officer (CEO), medical director) 2. Senior clinical staff (e.g. chief physician, chief of nursing) 3. Other facility management (e.g. biosafety, waste, those tasked with addressing water, sanitation and health (WASH)) |
| 3B | Did the committee meet in the past 12 months?  **Choose one answer** | 1. No **(skip to 4)** 2. Yes |
| 3C | Did you keep notes for the IPC committee meetings conducted in the past 12 months?  **Choose one answer**  **If yes, please ask for a copy of meeting notes from all the meetings conducted during the past 12 months and verify the answer.** | 1. No 2. Yes, for some 3. Yes, for all |
| **4** | When was the last time someone from the facility management, led or participated in a meeting to discuss IPC-related objectives, targets and challenges?  **Choose one answer**  **Please verify the answer based on the available documents.** | 1. Never 2. More than 3 months ago 3. Within the past 3 months |
| **5** | Does your facility have microbiological laboratory support for routine day-to-day use?  **Choose one answer** | 1. No 2. No, but the facility has access to a clinical laboratory at another site 3. Yes, an on-site laboratory is available |

| **Core component 2: Infection Prevention and Control (IPC) guidelines** | | | |
| --- | --- | --- | --- |
| **#** | | **Question** | **Answer** |
| **6.** | | Does your organization have any IPC guidelines, standard operating procedures (SOP) or internal rules, developed and approved at your organization? | 1. No **(skip to 7)** 2. Yes |
| **7.** | | Which guidelines or general SOPs are available at the facility?  **SELECT ALL THAT APPLY**  **Please verify the answer based on the available documents.** | 1. Standard precautions (universal precaution measures) 2. Hand hygiene 3. Transmission-based precautions 4. Outbreak management and preparedness 5. Prevention of surgical site infection 6. Prevention of vascular catheter-associated bloodstream infections 7. Prevention of hospital-acquired pneumonia ([HAP]; all types of HAP, including (but not exclusively) ventilator-associated pneumonia 8. Prevention of catheter-associated urinary tract infections 9. Prevention of transmission of multidrug-resistant (MDR) pathogens 10. Disinfection and sterilization 11. Health care worker protection and safety 12. Injection safety 13. Waste management 14. Antibiotic stewardship |
| **8** | Which statement best describes the process you use in this facility to develop or adapt IPC Guidelines?  **Choose one answer** | | 1. Facility uses international guidelines that have not been adapted to facility context **(Skip to 10)** 2. Facility uses national guidelines **(Skip to 10)** 3. Facility develops its own guidelines based on the national or international guidelines |
| **9** | | Who is involved in the development and/or adaptation of the IPC guidelines and SOPs at your organization?  **SELECT ALL THAT APPLY** | 1. IPC staff 2. Senior leadership 3. Clinical staff 4. Facility safety staff (ex., engineering service staff, water supply staff) 5. Quality management staff |
| **10** | | Please describe the process you use to train HCW on IPC guidelines when they are issued/updated (use an example from the last updated SOP and guidelines)?  Choose one answer  **Please ask to see any training notes and/or list of participants and agenda from the last training.** | 1. The facility does not have any structured or systematic process to train HCW on IPC guidelines 2. Newly developed or revised SOPs were posted on information boards for HCW to review 3. Oral instructions on newly developed or revised SOPs were provided to HCW during routine staff meetings 4. Some oral instructions included interactive sessions (online or on site) 5. All oral instructions included interactive sessions (online or on site) |

| **Core component 3: Infection Prevention and Control (IPC) trainings** | | |
| --- | --- | --- |
| **11** | Does this facility keep track of which clinical and non-clinical staff have been trained in IPC, when and in which topics?  **Choose one answer**  **Please verify the answer based on the available documents. If there is no documented proof that they track training participation, please mark No** | 1. No 2. Yes |
| **12** | Did this facility conduct any IPC trainings for clinical staff and others having contact with patients or wards during the past 12 months?  **SELECT ALL THAT APPLY**  **Please ask for copies of all training materials (agenda and list of participants) from all IPC-related trainings conducted during the past 12 months** | 1. No IPC trainings conducted at this facility **(Skip to 13)** 2. Yes, for health care workers (clinical staff) 3. Yes, for non-clinical staff with access to patients or wards at your facility (e.g. cleaners, auxiliary service staff, administrative and managerial staff) 4. Yes, for family members, other care-givers or visitors |
| **12A** | Did IPC trainings for clinical and non-clinical staff conducted during the past 12 months include interactive training sessions (simulations and/or bedside trainings)?  **Choose one answer**  **Please verify the answer based on the available documents. If not verified, then mark a different answer** | 1. No, interactive trainings only included written information and/or oral instructions and/or e-learning 2. Some trainings also included interactive sessions 3. All trainings included interactive sessions |
| **12B** | Who led IPC trainings for clinical and non-clinical staff conducted during the past 12 months?  **SELECT ALL THAT APPLY**  **Please verify the answer based on the available documents.** | 1. External trainers from outside the facility 2. IPC team members 3. Non-IPC personnel |
| **12C** | How did you assess the effectiveness of IPC trainings conducted during the past 12 months?  **SELECT ALL THAT APPLY**  **Please verify the answer based on the available documents.** | No assessment  Pre/post test  Post-training survey for participants   1. Compliance monitoring of IPC practices |
| **12D** | Did the facility conduct any specific IPC training for patients or family members to minimize the potential for health care-associated infections during the past 12 months?  (for example, staff provides special education for immunosuppressed patients, patients with invasive devices, patients with multidrug-resistant infections)  **Please verify the answer based on the available documents – ex., example of patient education (or a parent, a guardian for pediatric patients)** | No   1. Yes, regular and routine (scheduled) group trainings or individual trainings (not confirmed by any documentation) 2. Yes, documented regular and routine (scheduled) group trainings or individual trainings (not confirmed by any documentation) |
| **13** | In general, which statement best describes when IPC training for clinical staff at your facility is delivered?  **SELECT ALL THAT APPLY**  **Please verify the answer based on the available documents (training registers, tracking sheets, prikazes, etc).** | 1. All new HCWs are trained as part of new employee orientation 2. Ongoing regular training at least annually, but not mandatory 3. Ongoing mandatory training at least annually (not confirmed by any documentation) 4. Ongoing mandatory training at least annually (verified by documentation) 5. None of the above |
| **14** | In general, which statement best describes when IPC training for non-clinical staff in your facility is delivered?  **SELECT ALL THAT APPLY**  **Please verify this answer based on the available documents (training registers, tracking sheets, prikazes, etc).** | 1. All new non-clinical staff are trained as part of new employee orientation 2. Ongoing regular training at least annually, but not mandatory 3. Ongoing mandatory training at least annually (not confirmed by any documents) 4. Ongoing mandatory training at least annually (verified by documentation) 5. None of the above |
| **15** | Do clinical trainings conducted at your facility during the past 12 months for physicians working in specialty areas (for example, surgery or anesthesiology) include IPC?  **For example, if there is a line insertion training, would HH and skin prep standards be embedded in it, not just taught separately as IPC training?**  **Choose one answer**  **Please verify the answer based on the available documents.** | 1. No clinical trainings for specialists conducted at the facility during the past 12 months 2. clinical trainings for specialists were conducted during the past 12 months, but IPC was not included 3. Yes, in some trainings 4. Yes, in all trainings |

| **Core component 4: Health care-associated infection (HAI) surveillance** | | | |
| --- | --- | --- | --- |
| **#** | | **Question** | **Answer** |
| **16** | | Does this facility conduct HAI surveillance?  **Choose one answer**  **Please ask for all the available HAI surveillance documents** | 1. No **(skip to 26)** 2. Yes |
| **17** | | Do you have personnel responsible for surveillance activities? | 1. No **(skip to 18)** 2. Yes, only IPC staff 3. Yes, IPC staff, clinical staff, and if available – microbiology staff |
| **17A** | | Have the professionals responsible for surveillance activities been trained in basic epidemiology, surveillance and IPC (that is, do these people have the capacity to oversee surveillance methods, data management and interpretation)? | 1. No 2. Yes |
| **18** | Does HAI surveillance include any of the following?  **Please verify the answers based on the available documents.**  **SELECT ALL THAT APPLY** | | 1. List of priority healthcare associated infections which are major causes of morbidity and mortality in the facility   **If prioritization process is not described in the document, please ask to describe the process used to identify infections which are major causes of morbidity and mortality in the facility**   1. standardized case-definitions (defined numerator and denominator) 2. standardized data collection methods 3. processes to review data quality (for example, assessment of case report forms, review of microbiology results, denominator determination, etc.) 4. Clearly defined roles and responsibilities of staff involved in surveillance 5. Annual work plan and schedule 6. None of the above |
| **19** | For which infection types do you conduct surveillance at your facility?  **SELECT ALL THAT APPLY**  **Please verify the answers based on the available documents.** | | 1. Surgical site infections 2. Device-associated infections (for example, catheter-associated urinary tract infections, central line-associated bloodstream infections, peripheral-line associated bloodstream infections, ventilator-associated pneumonia) 3. Clinically-defined infections (for example, definitions based only on clinical signs or symptoms in the absence of microbiological testing) 4. Infections or colonization caused by multidrug-resistant pathogens (non-susceptibility to at least one agent in three or more antimicrobial categories) 5. Infections in targeted vulnerable patient populations (for example, neonates, intensive care unit, immunocompromised, burn patients) 6. Local priority epidemic-prone infections (for example, SARS-CoV-2, norovirus, influenza, tuberculosis) 7. Infections that may affect health care workers (for example, hepatitis B or C, HIV, influenza) |
| **20** | What data sources do you use for your HAI surveillance?  **SELECT ALL THAT APPLY**  **Please verify the answers based on the available documents.** | | 1. Discharge diagnosis data 2. Voluntary notification from physicians or nurses 3. Ward-based assessments (e.g., chart review, discussion with nurses or physicians, patient exam) 4. Laboratory-based assessment (e.g., review of blood cultures) 5. None of these types of surveillance |
| **21** | | Do you have informatics / IT support to conduct your surveillance  (for example, equipment, mobile technologies, electronic health records)? | 1. No 2. Yes |
| **22** | | Do you have microbiology and laboratory capacity to support surveillance?  Choose one answer | 1. No 2. Laboratory is able to differentiate between gram positive and gram negative strains but cannot identify the pathogen 3. The laboratory can identify most pathogens (e.g. isolate identification) 4. The laboratory can identify most pathogens and antimicrobial susceptibility patterns |
| **23** | During the past 12 months, how were your HAIs surveillance data shared with facility staff?  **SELECT ALL THAT APPLY**  **Please ask for any available reports or staff meetings to verify the answers** | | 1. Not shared with facility staff **(skip to 25)** 2. Written reports 3. Oral updates 4. Presentation |
| **24** | How often, do you provide up-to-date HAIs surveillance information?  **Choose one answer for questions**  **Please verify the answers based on the available documents** | | |
| **24A** | Clinical staff? | | 1. Never 2. Quarterly 3. Half-yearly 4. Annually 5. Periodically but no regular schedule 6. Monthly |
| **24B** | Non-clinical staff that have direct contact with patients | | 1. Never 2. Quarterly 3. Half-yearly 4. Annually 5. Periodically but no regular schedule 6. Monthly |
| **24C** | Clinical managers/heads of department? | | 1. Never 2. Quarterly 3. Half-yearly 4. Annually 5. Periodically but no regular schedule 6. Monthly |
| **24D** | IPC committee? | | 1. Never 2. Quarterly 3. Half-yearly 4. Annually 5. Periodically but no regular schedule 6. Monthly |
| **24E** | Non-clinical management? | | 1. Never 2. Quarterly 3. Half-yearly 4. Annually 5. Periodically but no regular schedule 6. Monthly |
| **25** | Are HAIs surveillance data used to make unit/facility- specific plans for the improvement of IPC practices?  **Choose one answer**  **Please ask to provide examples and verify the answer** | | 1. No 2. Yes |
| **26** | How often do you analyze and report antimicrobial drug resistance data?  **Choose one answer** | | 1. Never 2. Quarterly 3. Half-yearly 4. Annually 5. Periodically but no regular schedule 6. Monthly |

| **Core component 5: Multimodal strategies for implementation of infection prevention and control (IPC) interventions** |
| --- |

| **#** | **Question** | **Answer** |
| --- | --- | --- |
| **HAND HYGIENE** | | |
| **System change** | | |
| **27.** | Is there dedicated/available budget for the continuous procurement of hand hygiene products (e.g. alcohol-based handrubs)?  **Choose one answer**  **Please verify the answers based on the available documents** | 1. No 2. Yes |
| **28.** | How easily available is alcohol-based handrub containing either 75% of isopropanol, or 80% ethanol in the facility?  Point-of-care products should be accessible without having to leave the patient zone (ideally within arms reach of the health-care worker or within 2 meters). | 1. Not available 2. Yes, stations present, but supplies are not always available OR not at each point of care OR have less than 75% of isopropanol, or 80% ethanol 3. Yes, always available |
| **29** | Is soap available at each sink? | 1. No 2. Yes |
| **30** | Are single-use towels available at each sink? | 1. No 2. Yes |
| **Training and education** | | |
| **31** | How frequently do health-care workers receive training regarding hand hygiene in your facility?  **Please verify the answers based on the available documents** | 1. Never (Skip to 31) 2. At least once or more, but without any particular schedule 3. Regular training for medical and nursing staff, or all professional categories (at least annually) 4. Mandatory training for all professional categories at commencement of employment, then ongoing regular training (at least annually) |
| **31A** | Is a process in place to confirm that all health-care workers complete this training? | 1. No 2. Yes |
| **31B** | How do health-care workers receive training on hand hygiene in your facility? | 1. Only written information and/or oral instruction and/or e-learning 2. Training include interactive training sessions (includes simulation and/or bedside training) |
| **32** | Is there at least one medical staff or nursing staff trained in Infection Control or Infectious Diseases, whose tasks formally include dedicated time for staff training on hand- hygiene? | 1. No 2. Yes |
| **Evaluation and Feedback** | | |
| **33** | During the past 12 months, how often did you conduct direct observation of hand hygiene compliance performed using the WHO Hand Hygiene Observation tool (or similar observation tools)  **If monitoring tools are not available (checklist and schedule), but facility staff claim to conduct monitoring frequently, mark** “**Periodically but no regular schedule”** | 1. Not conducted 2. Periodically but no regular schedule 3. At least monthly 4. At least every 3 months 5. At least 2 times per year 6. Once a year |
| **34** | During the past 12 months, how often did you conduct monitoring/auditing consumption/usage of alcohol-based hand rub or soap  **If monitoring tools are not available (checklist and schedule), but facility staff claim to conduct monitoring frequently, mark** “**Periodically but no regular schedule”** | 1. Not conducted 2. Periodically but no regular schedule 3. At least monthly 4. At least every 3 months 5. At least 2 times per year 6. Once a year |
| **35** | Health care worker knowledge of which of the following topics assessed at least annually (e.g. after education sessions)?  **If there are no documents to confirm the audits, indicate “No”**  **Please verify the answers based on the available documents** | 1. Not assessed 2. The indications for hand hygiene 3. The correct technique for hand hygiene |
| **36** | How often do you provide feedback of data related to hand hygiene indicators with demonstration of trends over time given to Health-care workers?  **Please verify the answers based on the available documents** | 1. Never 2. Irregularly 3. Annually 4. At least every 6 months |
| **Reminders at Workplace** | | |
| **37** | Is hand hygiene promotion undertaken by displaying and regularly updating posters on hand hygiene? | 1. No **(Skip to 36)** 2. Yes, visual reminders and posters to promote or raise awareness of hand. hygiene 3. Yes, additional methods/initiatives to improve team communication across units and disciplines (for example, by facilitating multidisciplinary rounds, thematical conferences) |
| **37A** | Visible reminders, posters, or other tools to promote or raise awareness of hand hygiene | 1. Available at some hand hygiene stations 2. Available at all hand hygiene stations |
| **37B** | How frequently does a systematic audit of all posters for evidence of damage occur, with replacement as required?  **Please verify the answers based on the available documents** | 1. Never 2. At least annually 3. Every 2-3 months |
| **Institutional Safety Climate for Hand Hygiene** | | |
| **38** | Do you have a dedicated hand hygiene team to conduct active promotion and implementation of optimal hand hygiene practice in your facility?  **Please verify the answers based on the available documents** | 1. No (Skip to 37) 2. Yes |
| **38A** | How often do they meet to discuss hand hygiene promotion and implementation?  **Please verify the answers based on the available documents** | 1. Never 2. Irregularly 3. Annually 4. Quarterly 5. Monthly |
| **39** | Has a clear plan for the promotion of hand hygiene throughout the entire facility for the 5 May (Save Lives Clean Your Hands Annual Initiative) been established?  **Please verify the answers based on the available documents** | 1. No 2. Yes |
| **40** | Are your quality improvement staff involved in hand hygiene promotion and implementation activities?  Choose one answer | 1. NO Quality improvement staff exist at the facility 2. No 3. Yes |
| **41** | Do managers/leaders (head of the hospital, chief clinician and head of nursing) show visible support and act as champions and role models, promoting an adaptive approach and strengthening a culture that supports hand hygiene  **Please provide examples** | 1. No 2. Yes |
| **42** | Do facility staff (clinical and non-clinical) are empowered to routinely participate in hand hygiene improvement activities  **Please provide examples** | 1. No 2. Yes |
| **INJECTION SAFETY** | | |
| **System change** | | |
| **43** | Is there dedicated/available budget for the continuous procurement of safe injection equipment (single-use injection devices, gloves, sharps containers, etc)?  **Choose one answer**  **Please verify the answers based on the available documents** | 1. No 2. Yes |
| **43A** | Functional waste collection containers for non-infectious (general) waste, infectious waste and, sharps waste at all waste generation points? | 1. No bins or separate sharps disposal 2. Separate bins present but lids missing or more than 3/4 full;   or two bins (instead of three);  or bins at some but not all waste generation points.   1. Yes, all three containers |
| **Training and education** | | |
| **44** | How frequently do health-care workers receive training regarding injection safety in your facility?  **Please verify the answers based on the available documents** | 1. Never **(Skip to 43)** 2. At least once or more, but no regular schedule 3. Regular training for medical and nursing staff, or all professional categories (at least annually) 4. Mandatory training for all professional categories at commencement of employment, then ongoing regular training (at least annually) |
| **44A** | Is a process in place to confirm that all health-care workers complete this training? | 1. No 2. Yes |
| **44B** | How do health-care workers receive training regarding injection safety in your facility? | 1. Only written information and/or oral instruction and/or e-learning 2. Training include interactive training sessions (includes simulation and/or bedside training) |
| **45** | Is there at least one medical staff or nursing staff trained in Infection Control or Infectious Diseases, whose tasks formally include dedicated time for staff training on injection safety? | 1. No 2. Yes |
| **Evaluation and Feedback** | | |
| **46** | During the past 12 months, how often did you conduct direct observation of injection safety using WHO Injection Safety Assessment Tool (or similar observation tools)  **If monitoring tools are not available (checklist and schedule), but facility staff claim to conduct monitoring frequently, mark** “**Periodically but no regular schedule”** | 1. Not conducted (skip to 45) 2. Periodically but no regular schedule 3. At least monthly 4. At least every 3 months 5. At least 2 times per year 6. Once a year |
| **47** | During the past 12 months, how often did you conduct assessment of health care worker knowledge of injection safety principles?  **Please verify the answers based on the available documents**  **If monitoring tools are not available (checklist and schedule), but facility staff claim to conduct monitoring, mark** “**Periodically but no regular schedule”** | 1. Not conducted 2. Periodically but no regular schedule 3. At least monthly 4. At least every 3 months 5. At least 2 times per year 6. Once a year |
| **48** | How often do you provide feedback of data related to injection safety indicators with demonstration of trends over time given to Health-care workers?  **Please verify the answers based on the available documents** | 1. Never 2. Irregularly 3. Annually 4. At least every 6 months |
| **Reminders at Workplace** | | |
| **49** | Is injection safety promotion undertaken by displaying and regularly updating posters, other visual materials and other initiatives? | 1. No **(Skip to 48)** 2. Yes, visual reminders and posters to promote or raise awareness of injection safety 3. Yes, additional methods/initiatives to improve team communication across units and disciplines (for example, by facilitating multidisciplinary rounds, thematical conferences) |
| **49A** | Visible reminders, posters, or other tools to promote or raise awareness of injection safety | 1. Available at some units/wards/departments 2. Available at all units/wards/departments |
| **49B** | How frequently does a systematic audit of all posters for evidence of damage occur, with replacement as required?  **Please verify the answers based on the available documents** | 1. Never 2. At least annually 3. Every 2-3 months |
| **Institutional Safety Climate** | | |
| **50** | Do you have a dedicated injection safety team to conduct active promotion and implementation of injection safety practices in your facility?  **Please verify the answers based on the available documents** | 1. No **(Skip to 49)** 2. Yes |
| **50A** | How often do they meet to discuss injection safety promotion and implementation?  **Please verify the answers based on the available documents** | 1. Never 2. Irregularly 3. Annually 4. Quarterly 5. Monthly |
| **51** | Are your quality improvement staff involved in hand hygiene promotion and implementation activities?  Choose one answer | 1. NO Quality improvement staff exist at the facility 2. No 3. Yes |
| **52** | Do managers/leaders (head of the hospital, chief clinician and head of nursing) show visible support and act as champions and role models, promoting an adaptive approach and strengthening a culture that supports injection safety  **Please provide examples** | 1. No 2. Yes |
| **53** | Do facility staff (clinical and non-clinical) are empowered to participate in injection safety improvement activities  **Please provide examples** | 1. No 2. Yes |

| **Core component 6: Monitoring / audit of IPC practices and feedback** | | | |
| --- | --- | --- | --- |
| **#** | **Question** | | **Answer** |
| **54** | | Does this facility have an internal IPC monitoring/audit plan with any of the following?  **SELECT ALL THAT APPLY**  **Please verify the answer based on the available documents.** **Only select options that were verified by the document review.** | 1. No facility monitoring/audit plan available 2. Yes, with clear goals and objectives 3. Yes, with tools to collect data in a systematic way (for example checklists) 4. Yes, with clearly defined roles and responsibilities 5. Yes, with work plan or schedule |
| **55** | | When was the last time there was an internal IPC monitoring/audit to assess compliance of any IPC practices at your facility?  **Choose one answer**  **Please verify the answer based on the available documents** | 1. Within the past 3 months 2. Within the past 6 months 3. Within the past 12 months 4. More than 12 months ago or never |
| **56** | | Did IPC staff document in any form implementation of monitoring/audits conducted within the past 12 months documented?  **Please ask for a copy of all the available IPC monitoring/audit reports conducted during the past 12 months** | 1. No 2. Yes |
| **57** | | During the past 12 months, how often did you conduct monitoring/auditing of different IPC practices aside from hand hygiene and injection safety that we have already discussed earlier?  **Choose one answer for each of the following questions**  **Please verify the answers based on the available documents.** | |
| **57A** | | Waste management  **If monitoring tools are not available (checklist and schedule), but facility staff claim to conduct monitoring frequently, mark** “**Periodically but no regular schedule”** | 1. Not conducted 2. Periodically but no regular schedule 3. At least monthly 4. At least every 3 months 5. At least 2 times per year 6. Once a year |
| **57B** | | Cleaning of the ward environment  **If monitoring tools are not available (checklist and schedule), but facility staff claim to conduct monitoring frequently, mark** “**Periodically but no regular schedule”** | 1. Not conducted 2. Periodically but no regular schedule 3. At least monthly 4. At least every 3 months 5. At least 2 times per year 6. Once a year |
| **57C** | | Disinfection and sterilization of medical equipment/instruments  **If monitoring tools are not available (checklist and schedule), but facility staff claim to conduct monitoring frequently, mark** “**Periodically but no regular schedule”** | 1. Not conducted 2. Periodically but no regular schedule 3. At least monthly 4. At least every 3 months 5. At least 2 times per year 6. Once a year |
| **57D** | | Transmission-based precautions, isolation and cohorting (grouping) of patients to prevent the spread of multidrug resistant organisms (MDRO)  **If monitoring tools are not available (checklist and schedule), but facility staff claim to conduct monitoring frequently, mark** “**Periodically but no regular schedule”** | 1. Not conducted 2. Periodically but no regular schedule 3. At least monthly 4. At least every 3 months 5. At least 2 times per year 6. Once a year |
| **57E** | | Consumption/usage of antimicrobial agents  **If monitoring tools are not available (checklist and schedule), but facility staff claim to conduct monitoring frequently, mark** “**Periodically but no regular schedule”** | 1. Not conducted 2. Periodically but no regular schedule 3. At least monthly 4. At least every 3 months 5. At least 2 times per year 6. Once a year |
| **57F** | | Intravascular catheter insertion and/or care  **If monitoring tools are not available (checklist and schedule), but facility staff claim to conduct monitoring frequently, mark** “**Periodically but no regular schedule”** | 1. Not conducted 2. Periodically but no regular schedule 3. At least monthly 4. At least every 3 months 5. At least 2 times per year 6. Once a year |
| **57G** | | Wound dressing change  **If monitoring tools are not available (checklist and schedule), but facility staff claim to conduct monitoring frequently, mark** “**Periodically but no regular schedule”** | 1. Not conducted 2. Periodically but no regular schedule 3. At least monthly 4. At least every 3 months 5. At least 2 times per year 6. Once a year |
| **58** | | During the past 12 months, how did you share results of these internal monitoring /auditing of IPC practices with the following facility staff listed in questions 26A-E?  **SELECT ALL THAT APPLY** | |
| **58A** | | Clinical staff? | 1. Did not share 2. Shared orally during staff meetings 3. Shared in a form of a written report |
| **58B** | | Non-clinical staff that have direct contact with patients? | 1. Did not share 2. Shared orally during staff meetings 3. Shared in a form of a written report |
| **58C** | | Clinical managers/heads of department? | 1. Did not share 2. Shared orally during staff meetings 3. Shared in a form of a written report |
| **58D** | | IPC committee? | 1. Did not share 2. Shared orally during staff meetings 3. Shared in a form of a written report |
| **58E** | | Non-clinical management (CEO, administration, board)? | 1. Did not share 2. Shared orally during staff meetings 3. Shared in a form of a written report |
| **59** | | During the past 12 months, how often were your monitoring results used to make unit/facility- specific plans for the improvement of IPC practices?  **Choose one answer**  **If yes, please ask to provide examples. Please verify the answers based on the examples provided.** | 1. Always 2. Sometimes 3. Never |
| **60** | Do you think your organization’s culture allows discussion of monitoring and feedback of IPC processes and indicators in a constructive way, avoiding individual blame and punishment?  Are monitoring and feedback of IPC processes and indicators performed in a “blame-free” institutional culture aimed at improvement and behavioural change? | | 1. No 2. Yes |

| **Core component 7: Workload, staffing and bed occupancy** | | |
| --- | --- | --- |
| **#** | **Question** | **Answer** |
| **61** | Is a system in place to respond to situations when staffing levels are deemed to be too low?  In other words, if staffing schedule needs revision and some wards need more staff, does the organization allow such a change.  **Provide an example in the last 12 months** | 1. No 2. Yes |
| **62** | Is a system in place in your facility to assess and respond when adequate bed capacity is exceeded?  **Provide an example in the last 12 months**  Choose one answer | 1. No 2. Yes, this is the responsibility of the head of department 3. Yes, this is the responsibility of the hospital administration/ management |
| **62A** | Any patients in beds outside of the room (in the corridor) | 1. No 2. Yes |
| **62B** | More than 1 meter between patient beds? | 1. No 2. Yes, but not in all departments 3. Yes, for all units including pediatrics and emergency |

| **Core component 8: Built environment, materials and equipment for IPC at the facility level** | | |
| --- | --- | --- |
| **#** | **Question** | **Answer** |
| **63** | How much stock of the following **Hand hygiene supplies** do you have at your facility at the time of this assessment? | |
| **63A** | Alcohol-based hand rub | 1. No stock 2. Sufficient for less than 1 week 3. Sufficient for 1 week or more |
| **63B** | Soap | 1. No stock 2. Sufficient for less than 1 week 3. Sufficient for 1 week or more |
| **63C** | Disposable or reusable towels | 1. No stock 2. Sufficient for less than 1 week 3. Sufficient for 1 week or more |
| **63D** | Veronica buckets with functional taps, lids and basin for collecting used handwashing water (If functional sinks are not available in registration or waiting areas) | 1. No stock 2. Sufficient for less than 1 week 3. Sufficient for 1 week or more |
| **64** | How much stock of the following **cleaning supplies** do you have at your facility at the time of this assessment? | |
| **64A** | Neutral detergent, liquid or powdered soap | 1. No stock 2. Sufficient for less than 1 week 3. Sufficient for 1 week or more |
| **64B** | Cleaning cloths | 1. No stock 2. Sufficient for less than 1 week 3. Sufficient for 1 week or more |
| **64C** | Mops currently available | 1. No stock 2. Sufficient for less than 1 week 3. Sufficient for 1 week or more |
| **64D** | Portable buckets (for mopping and surface cleaning solutions) currently available | 1. No stock 2. Sufficient for less than 1 week 3. Sufficient for 1 week or more |
| **64E** | Hospital-grade disinfectants (e.g., ethanol (70-90%, 0.5% sodium hypochlorite) | 1. No stock 2. Sufficient for less than 1 week 3. Sufficient for 1 week or more |
| **65** | How much stock of the following **PPE supplies** do you have at your facility at the time of this assessment? | |
| **65A** | Non-sterile gloves | 1. No stock 2. Sufficient for less than 1 week 3. Sufficient for 1 week or more |
| **65B** | Gowns | 1. No stock 2. Sufficient for less than 1 week 3. Sufficient for 1 week or more |
| **65C** | Aprons | 1. No stock 2. Sufficient for less than 1 week 3. Sufficient for 1 week or more |
| **65D** | Eye protection (face shields or goggles) | 1. No stock 2. Sufficient for less than 1 week 3. Sufficient for 1 week or more |
| **65E** | Medical masks | 1. No stock 2. Sufficient for less than 1 week 3. Sufficient for 1 week or more |
| **65F** | N95, FFP2, or equivalent respirators | 1. No stock 2. Sufficient for less than 1 week 3. Sufficient for 1 week or more |

| **General questions**  Before we go around the facility, we would like to ask you a couple of more general questions | |
| --- | --- |
| **66** | What do you consider the top challenges to implementing IPC at this facility? |
| **67** | What would be your recommendations to improve IPC in this facility? |
| **68** | What do you consider the top challenges to implementing HAI surveillance? |

## PART 2 – Observation checklist

The checklist should be completed by the assessment team based on direct observations during a random walk around the facility. The assessment team will need to verify some of the answers to the questions asked during Part 1 of the interview. It is recommended that the random walk will include entrance and patient waiting areas, visits to one ICU (if available) and a typical ward (department). In case of facilities that have pediatric and adult wards, the team needs to visit one adult ward and one pediatric ward. In each ward, the team must check a minor procedures room and 2-5 patient rooms.

Please check the availability of the following:

| **#** | **Question** | **Answer** |
| --- | --- | --- |
| **1** | Patients and staff have dedicated entrances clearly labeled  ***Observe; note any locations that are not well marked.*** | 1. No 2. Yes |
| **2** | Signs or posters directing patients with COVID-19 symptoms to proceed directly to the registration desk are posted at all facility entrances  ***Locate all entrances to verify that signs or posters are posted. If no, list in the comments, list the areas where signs or posters are not available.*** | 1. No 2. Yes |
| **3** | There are signs or other materials visible at the entrances to the facility instructing visitors not to visit if they have fever or symptoms of COVID-19 | 1. No 2. Yes |
| **4** | All patients access the facility through the screening > waiting room > triage > wards; the flow is rational, clear, and properly labeled  ***Follow the flow of a patient; make schematic to identify weak points*** | 1. No 2. Yes |
| **5** | All staff access the facility through staff entrance > syndromic surveillance (questionnaire/assessment) > changing room > staff area; the flow is rational, clear and properly labelled  ***Follow the flow of a staff member; make schematic to identify weak points*** | 1. No 2. Yes |
| **6** | All visitors access the facility through a dedicated controlled and equipped (hand hygiene station) entrance including a screening station  ***Follow the flow of a visitor; make schematic to identify weak points*** | 1. No 2. Yes |
| **7** | All visitors are screened for COVID-19 symptoms  Check facility logbook or observe screening process | 1. No 2. Yes |
| **8** | A physical barrier is in place between staff and patients presenting to the registration desk (for example, a plastic/glass window or table providing at least 1-meter~~*~~ separation)  ***Check registration desk to verify physical barrier is present*** | 1. No 2. Yes |
| **9** | Facility has a separate COVID-19 waiting area for suspected COVID-19 patients | 1. No (SKIP to 8) 2. Yes |
| **9А** | The waiting area is well ventilated (natural or mechanical) | 1. No 2. Yes |
| **9B** | Benches, chairs, or other seating in the separate COVID-19 waiting area have at least 1-meter~~*~~ distance between them | 1. No 2. Yes |
| **9C** | Functional hand hygiene stations are available near the registration desk and in the waiting area | 1. No 2. Yes |
| **9D** | The following items are available at all times near the registration desk and/or in the waiting area: | ☐ Masks  ☐ Tissues  ☐ Waste bin |
| **9E** | Dedicated toilets are available for patients in the separate waiting area | 1. No 2. Yes |
| **10** | Single rooms with doors are available for physical evaluation of patients with COVD-19 symptoms | 1. No 2. Yes |
| **11** | If single rooms are not available, a well-ventilated, private area away from other patients is available for conducting physical evaluations | 1. No 2. Yes |
| **12** | COVID-19 screening forms and/or flow chart are available for HCWs evaluating patients in the separate area | 1. No 2. Yes |
| **13** | HCWs conducting physical evaluations of patients with respiratory symptoms have access to gowns, gloves, face masks, and eye protection | 1. No 2. Yes |
| **14** | High touch surfaces in waiting areas are cleaned at least twice a day (surfaces should be cleaned and disinfected using an approved disinfectant product)  ***Ask cleaning staff how often they clean the area; See documentation in cleaning log book if available*** | 1. No 2. Yes |
| **15** | Visible reminders, posters, or other tools to promote or raise awareness of hand hygiene and COVID-19 related precautions and requirements  **Choose one answer** | 1. Not available 2. Available at some hand hygiene stations observed 3. Available at all hand hygiene stations observed |
| **16** | Visible reminders, posters, or other tools to promote or raise awareness of injection safety  **Choose one answer** | 1. Not available 2. Available at some units/wards/departments observed 3. Available at all units/wards/departments observed |
| **17** | Any patients in beds outside of the room (in the corridor) | 1. No 2. Yes |
| **18** | More than 1 meter between patient beds? | 1. No 2. Yes, but not in all departments 3. Yes, for all units including pediatrics and emergency |
| **19** | How easily available is alcohol-based handrub containing either 75%, or isopropanol, or 80% ethanol in the facility?  *Point-of-care products should be accessible without having to leave the patient zone (ideally within arms reach of the health-care worker or within 2 meters).* | 1. Not available 2. Yes, stations present, but supplies are not always available OR not at each point of care 3. Yes, always available |
| **20** | Is soap available at each sink? | 1. No 2. Yes |
| **21** | Are single-use towels available at each sink? | 1. No 2. Yes |
| **22** | Functional waste collection containers for non-infectious (general) waste, infectious waste and, sharps waste at all waste generation points? | 1. No bins or separate sharps disposal 2. Separate bins present but lids missing or more than 3/4 full;   or two bins (instead of three);  or bins at some but not all waste generation points.   1. Yes, all three containers |
