# Supplementary material for: Core components of infection prevention and control programs at the facility level in Kazakhstan: key challenges and opportunities
Source: Antimicrob Resist Infect Control. 2023 Jun 22;12:59. doi: 10.1186/s13756-023-01264-6 (PMC10286477; doi:10.1186/s13756-023-01264-6)
Supplement: Supplementary file 2 — Additional file 2. Annex 2. [file 13756_2023_1264_MOESM2_ESM.docx]

Annex 2. Detailed assessment results (N=78)

| **Facility characteristics** | **Total   n (%)** | **Public (N=53)** | **Private**  **(N=25)** |
| --- | --- | --- | --- |
|  |  | **n (%)** | |
| **IPC program** |  |  |  |
| Had at least one designated IPC specialist | 78 (100) | 53 (100) | 25 (100) |
| Had at least one designated IPC specialist who had received formal IPC training | 59 (76) | 47 (89) | 12 (48) |
| Had more than one properly trained IPC team member | 31 (40) | 28 (53) | 3 (12) |
| IPC team included nurses trained in IPC | 48 (61) | 39 (74) | 9 (36) |
| Had an internal IPC policy (program), that included: | 71 (91) | 52 (98) | 19 (76) |
| specially allocated budget | 15 (19) | 10 (19) | 5 (20) |
| clearly defined KPIs | 5 (6) | 2 (4) | 3 (12) |
| clearly defined IPC workplan | 42 (54) | 28 (53) | 14 (56) |
| clearly defined objectives | 30 (38) | 22 (42) | 8 (32) |
| clearly defined objectives and future goals | 4 (5) | 4 (8) | 0 (0) |
| IPC objectives were based on surveillance and risks assessment | 9 (12) | 7 (13) | 2 (8) |
| Had an established multidisciplinary IPC committee | 74 (95) | 53 (100) | 21 (84) |
| IPC committee included senior leadership | 73 (94) | 51 (96) | 20 (80) |
| IPC committee included biosafety and WASH staff | 30 (38) | 26 (49) | 4 (16) |
| IPC committee met at least once in the past 12 months | 60 (77) | 44 (83) | 16 (64) |
| Had access to a microbiological lab | 75 (97) | 52 (98) | 24 (96) |
| in-house microbiological lab | 28 (36) | 24 (45) | 4 (16) |
| outsourced microbiological support | 48 (62) | 28 (53) | 20 (80) |
| **IPC guidelines** | |  |  |
| Implemented internal guidelines and/or SOPs developed by facility | 35 (45) | 25 (47) | 10 (40) |
| Used national guidelines | 37 (47) | 26 (49) | 11 (44) |
| Had any IPC guidelines and/or SOPs available, including | 72 (92) | 51 (96) | 21 (84) |
| Standard precautions (universal precaution measures) | 35 (45) | 21 (40) | 14 (56) |
| Hand hygiene | 70 (90) | 50 (94) | 20 (80) |
| Transmission-based precautions | 34 (44) | 22 (42) | 12 (48) |
| Outbreak management and preparedness | 30 (38) | 19 (36) | 11 (44) |
| Prevention of surgical site infection | 30 (38) | 22 (42) | 8 (32) |
| Prevention of vascular catheter-associated bloodstream infections | 20 (26) | 14 (26) | 6 (24) |
| Prevention of hospital-acquired pneumonia ([HAP]; all types of HAP, including (but not exclusively) ventilator-associated pneumonia | 11 (14) | 9 (17) | 2 (8) |
| Prevention of catheter-associated urinary tract infections | 20 (26) | 13 (25) | 7 (28) |
| Prevention of transmission of multidrug-resistant (MDR) pathogens | 8 (10) | 7 (13) | 1 (4) |
| Disinfection and sterilization | 69 (88) | 50 (94) | 19 (76) |
| Healthcare worker protection and safety | 52 (67) | 40 (75) | 12 (48) |
| Injection safety | 20 (26) | 40 (75) | 12 (48) |
| Waste management | 69 (88) | 49 (92) | 20 (80) |
| Antibiotic stewardship | 28 (36) | 21 (40) | 7 (28) |
| Screening of incoming patients for SARS-CoV-2 | 37 (47) | 29 (55) | 8 (32) |
| Conducted structured or systematic process to train HCW on IPC guidelines and/or new SOPs | | | |
| newly developed or revised SOPs were posted on information boards for HCW to review | 12 (15) | 11 (21) | 1 (4) |
| oral instructions on newly developed or revised SOPs were provided to HCW during routine staff meetings | 41 (53) | 30 (57) | 11 (44) |
| some oral instructions included interactive sessions (online or on site) | 7 (9) | 5 (9) | 2 (8) |
| all oral instructions included interactive sessions (online or on site) | 2 (3) | \| 0 (0) \| 2 (8) \| \| --- \| --- \| | 2 (8) |
| **IPC training** | |  |  |
| Conducted any IPC trainings in the past 12 months, including for: | | | |
| clinical staff | 63 (81) | 47 (89) | 16 (64) |
| non-clinical staff having contact with patients or wards | 25 (32) | 20 (38) | 5 (20) |
| family members, caregivers or visitors | 17 (22) | 12 (23) | 5 (20) |
| IPC training included interactive sessions (at least some trainings) | 34 (44) | 24 (45) | 10 (40) |
| Attendance of IPC trainings was monitored and recorded | 54 (69) | 40 (75) | 14 (56) |
| Conducted IPC training for all clinical staff as part of new employee orientation | 55 (71) | 40 (75) | 15 (60) |
| Conducted IPC training for all non-clinical staff as part of new employee orientation | 44 (56) | 36 (68) | 8 (32) |
| Required ongoing mandatory training at least annually for all clinical staff (documented & non-documented)) | 55 (71) | 38 (72) | 17 (68) |
| Required ongoing mandatory training at least annually for all non-clinical staff (documented & non-documented)) | 33 (42) | 24 (45) | 9 (36) |
| **HAI surveillance** | |  |  |
| Reported conducting HAI surveillance | 58 (74) | 44 (83) | 14 (56) |
| Had at least one component of a system for HAI surveillance, including: | | | |
| annual HAI surveillance work plan and schedule | 36 (46) | 28 (53) | 8 (32) |
| clearly defined roles and responsibilities of staff involved in HAI surveillance | 11 (14) | 9 (17) | 2 (8) |
| list of priority HAIs, which are major causes of morbidity and mortality in the facility | 10 (13) | 9 (17) | 1 (4) |
| processes to review data quality | 17 (22) | 14 (26) | 3 (12) |
| standardized case-definitions (defined numerator and denominator) | 9 (12) | 9 (17) | 0 (0) |
| standardized data collection methods | 11 (14) | 9 (17) | 2 (8) |
| surveillance system includes none of the above components | 13 (17) | 9 (17) | 4 (16) |
| Ever conducted analysis of AMR, including | 24 (31) | 19 (36) | 5 (20) |
| Quarterly | 5 (6) | 5 (9) | 0 (0) |
| Semiannually | 2 (3) | 2(4) | 0 (0) |
| Annually | 1 (1) | 1 (2) | 0 (0) |
| Periodically but no regular schedule | 12 (15) | 8 (15) | 4 (16) |
| **Multimodal strategies** | |  |  |
| ***Hand hygiene*** | |  |  |
| Dedicated/available budget for the continuous procurement of hand hygiene products | 68 (87) | 46 (87) | 22 (88) |
| Alcohol-based handrub containing either 75, or isopropanol, or 80 ethanol always available always and at each point of care (reported) | 8 (10) | 6 (11) | 2 (8) |
| Alcohol-based handrub containing either 75, or isopropanol, or 80 ethanol always available always and at each point of care (observed) | 8 (10) | 5 (9) | 3 (12) |
| Single-use towels available at each sink (reported) | 30 (38) | 21 (40) | 9 (36) |
| Single-use towels available at each sink (observed) | 26 (33) | 16 (30) | 10 (40) |
| Soap available at each sink (reported) | 48 (62) |  |  |
| Soap available at each sink (observed) | 45 (58) | 34 (64) | 14 (56) |
| Required hand hygiene training for all facility staff as part of new employee orientation and then ongoing mandatory training at least annually | 34 (44) | 24 (45) | 10 (40) |
| Had at least one medical staff or nursing staff trained in IPC or Infectious Diseases, whose tasks formally include dedicated time for staff training on hand hygiene | 16 (21) | 11 (21) | 5 (20) |
| During the past 12 months, at least once conducted direct observation of hand hygiene compliance performed using the WHO Hand Hygiene Observation tool (or similar observation tools), including: | 57 (73) | 41 (77) | 16 (64) |
| At least once a month | 19 (24) | 14 (26) | 5 (20) |
| At least once every quarter | 2 (3) | 2 (4) | 0 (0) |
| Periodically but no regular schedule | 36 (46) | 25 (47) | 11 (44) |
| During the past 12 months, at least once conducted monitoring of consumption/usage of alcohol-based hand rub or soap | 48 (61) | 31 (58) | 17 (68) |
| At least once a month | 10 (13) | 6 (11) | 4 (16) |
| At least once every quarter | 3 (4) | 1 (2) | 2 (8) |
| At least 2 times per year | 1 (1) | 1 (2) | 0 (0) |
| Periodically but no regular schedule | 34 (44) | 23 (43) | 11 (44) |
| At least annually assessed health care worker knowledge of indicators for hand hygiene | 30 (38) | 23 (43) | 7 (28) |
| At least annually assessed health care worker knowledge of the correct technique for hand hygiene | 50 (64) | 35 (66) | 15 (60) |
| *Provided feedback of data related to hand hygiene indicators with demonstration of trends over time given to healthcare workers* | 37 (47) | 24 (45) | 13 (52) |
| Used reminders, posters, or other tools to promote or raise awareness of hand hygiene | 72 (92) | 50 (94) | 22 (88) |
| Used additional methods/initiatives to improve team communication across units/specialties | 6 (8) | 4 (8) | 2 (8) |
| Managers/leaders show visible support and act as champions/role models | 68 (87) | 47 (89) | 21 (84) |
| Facility staff were empowered to participate in hand hygiene improvement activities | 39 (50) | 25 (47) | 14 (56) |
| ***Injection safety*** | |  |  |
| Dedicated/available budget for injection safety assurance | 66 (85) | 46 (87) | 20 (80) |
| Functional waste collection containers for non-infectious (general) waste, infectious waste and, sharps waste available at all waste generation points (reported) | 37 (47) | 27 (51) | 10 (40) |
| Functional waste collection containers for non-infectious (general) waste, infectious waste and, sharps waste available at all waste generation points (observed) | 28 (36) | 20 (38) | 8 (32) |
| Required injection safety training for all facility staff as part of new employee orientation and then ongoing mandatory training at least annually | 5 (6) | 4 (8) | 1 (4) |
| Had at least one medical staff or nursing staff trained in IPC or Infectious Diseases, whose tasks formally include dedicated time for staff training on injection safety | 2 (3) | 2 (4) | 0 (0) |
| During the past 12 months, conducted at least once direct observation of injection safety compliance performed using WHO Injection Safety Assessment Tool (or similar observation tools). | 14 (18) | 9 (17) | 5 (20) |
| At least annually assessed health care worker knowledge on injecting safety | 15 (19) | 11 (21) | 3 (12) |
| Provided feedback of data related to injection safety indicators with demonstration of trends over time given to healthcare workers | 6 (8) | 6 (11) | 0 (0) |
| Reminders, posters, or other visual tools used to promote or raise awareness of injection safety (reported) | 4 (5) | 4 (8) | 0 |
| Reminders, posters, or other visual tools used to promote or raise awareness of injection safety (observed) | 0 | 0 | 0 |
| Facility staff are empowered to participate in injection safety improvement activities | 19 (24) | 13 (25) | 6 (24) |
| Managers/leaders show visible support and act as champions/role models | 44 (56) | 31 (58) | 13 (52) |
| **IPC monitoring and audit** | |  |  |
| Had an IPC monitoring/audit plan, that included: | 23 (29) | 17 (32) | 6 (24) |
| Clear goals and objectives | 6 (8) | 4 (8) | 2 (8) |
| Clearly defined roles and responsibilities | 3 (4) | 3 (6) | 0 (0) |
| Monitoring results were used to make unit/facility-specific plans to improve IPC practices (At least sometimes) | 31 (40) | 18 (34) | 13 (52) |
| Tools to collect data in a systematic way | 14 (18) | 11 (21) | 3 (12) |
| Work plan or schedule | 15 (19) | 12 (23) | 3 (12) |
| Conducted at least one structured monitoring of any IPC practices during the past 12 months | 43 (55) | 31 (58) | 12 (48) |
| Documented results of monitoring conducted during the past 12 months | 38 (49) | 28 (53) | 10 (40) |
| Shared results (orally or in written) of the internal monitoring /auditing of IPC practices with all cadres of facility staff, including: |  |  |  |
| Clinical managers/heads of department | 64 (82) | 47 (89) | 17 (68) |
| Clinical staff (Orally or Written) | 55 (71) | 38 (72) | 17 (68) |
| IPC committee member (Orally or Written) | 56 (72) | 42 (79) | 14 (56) |
| Non-clinical management (CEO, administration, board) (Orally or Written) | 45 (58) | 30 (57) | 15 (60) |
| Non-clinical staff that have direct contact with patients (Orally or Written) | 24 (31) | 15 (28) | 9 (36) |
| Conducted structured monitoring of the following IPC practices at least once a month: | |  |  |
| Intravascular catheter insertion and/or care | 4 (5) | 3 (6) | 1 (4) |
| Transmission-based precautions, isolation and cohorting (grouping) of patients | 2 (3) | 1 (2) | 1 (4) |
| Waste management | 7 (9) | 7 (13) | 0 (0) |
| Wound dressing change | 2 (3) | 1 (2) | 1 (4) |
| **Workload, staffing and bed occupancy** |  |  |  |
| Had a system for responding to an increase in staff workload | 70 (90) | 48 (91) | 22 (88) |
| Had a system for assessing and responding to an exceeding bed capacity | 69 (88) | 50 (94) | 19 (76) |
| Adequate spacing (>1m) ensured between beds in all units (in all units) (reported) | 35 (45) | 20 (38) | 15 (60) |
| Adequate spacing (>1m) ensured between beds in all units (in all units) (observed) | 27 (35) | 15 (28) | 12 (48) |
| Patients never placed outside in corridor | 74 (95) | 49 (92) | 25 (100) |
| **Bult environment, materials and equipment for IPC** |  |  |  |
| Patients and staff had dedicated entrances clearly labeled | 30 (38) | 23 (43) | 7 (28) |
| All patients accessed the facility through the screening > waiting room > triage > wards; the flow is rational, clear, and properly labeled | 53 (68) | 38 (72) | 15 (60) |
| All staff accessed the facility through staff entrance > syndromic surveillance (questionnaire/assessment) > changing room > staff area; the flow is rational, clear and properly labelled | 46 (59) | 36 (68) | 10 (40) |
| All visitors accessed the facility through a dedicated controlled and equipped (hand hygiene station) entrance including a screening station | 61 (78) | 41 (77) | 20 (80) |
| Had a separate well-ventilated waiting area for patients with fever or respiratory symptoms | 48 (61) | 34 (64) | 14 (56) |
| Had a stock sufficient for 1 week or more for the following hand hygiene and cleaning supplies | |  |  |
| Buckets (for mopping and surface cleaning) | 74 (95) | 51 (96) | 23 (92) |
| Disinfectants for medical use (e.g. sodium hypochlorite) | 77 (99) | 52 (98) | 25 (100) |
| Mops | 77 (99) | 52 (98) | 25 (100) |
| Neutral detergent, liquid soap or soap powder | 75 (96) | 51 (96) | 24 (96) |
| Washcloths | 76 (97) | 51 (96) | 25 (100) |
| Alcohol based hand sanitizer | 66 (85) | 44 (83) | 22 (88) |
| Disposable towels | 57 (73) | 37 (70) | 20 (80) |
| Soap | 71 (91) | 47 (89) | 24 (96) |
| Had a stock sufficient for 1 week or more for the following PPE | |  |  |
| Aprons | 69 (88) | 46 (87) | 23 (92) |
| Eye protection (safety shields or goggles) | 71 (91) | 49 (92) | 22 (88) |
| Medical gowns | 73 (94) | 48 (91) | 25 (100) |
| Medical masks | 77 (99) | 52 (98) | 25 (100) |
| N95, FFP2 or similar respirators | 66 (85) | 47 (89) | 19 (76) |
| Non-sterile gloves | 75 (96) | 50 (94) | 25 (100) |
